# The impact of primary care access on autism spectrum disorder awareness in an underserved population

**DOI:** 10.3389/fpubh.2023.1250259

**Published:** 2023-12-18

**Authors:** Mackenzie T. O’Donnell, Randall B. Schmidt, Faith M. Butler

**Affiliations:** Department of Family Medicine and Community Health, University of Kansas Medical Center, Kansas City, KS, United States

**Keywords:** autism spectrum disorder, developmental screening, primary health care, physicians, primary care, free clinics, perception, safety-net providers

## Abstract

American Academy of Pediatrics (AAP) recommendations for Autism Spectrum Disorder (ASD) screening do not specifically address safety-net clinics, which provide multidisciplinary healthcare services to underserved patients. This project explored the potential for ASD screening in safety-net clinics by assessing parental perceived knowledge of ASD at JayDoc Free Clinic, a student-run safety-net clinic in Wyandotte County, Kansas. May through December 2022, patients who reported to be the parent of a minor received a demographic survey and a Likert-style questionnaire assessing perceived knowledge of ASD, including understanding the importance of ASD screening and ASD signs and symptoms. Responses were categorized into positive, negative, and unsure. Demographic variables included the minor’s primary care provider (PCP) status. Results were analyzed using bivariate analysis, with chi-square tests for significance (*p*-value ≤ 0.05). Of the 52 participants who completed at least one Likert response, 55.8% reported their child had a PCP. Responses were somewhat balanced with 44.2% positive for understanding the importance of ASD screening and 53.8% positive for understanding ASD signs and symptoms. For understanding the signs and symptoms of ASD, an unsure response (32.7% of responses) was statistically associated with a lack of PCP (*p* = 0.017). The balance of positive with negative and unsure responses could reflect lack of ASD knowledge and may relate to healthcare inaccessibility. This is consistent with the significant association between lack of PCP and unsure responses for understanding ASD signs and symptoms. ASD screening and education in safety-net clinics like JayDoc could be valuable, particularly for children without a PCP.

## 1 Introduction

Autism Spectrum Disorder (ASD) is a developmental disorder affecting social communication and interaction and accompanied by restricted repetitive behavior (1). With a prevalence of 1 in 59 children, early diagnosis and treatment of this disorder is crucial for children and their families (2). The American Academy of Pediatrics (AAP) advises ASD screening at the 18 and 24-month well-child checks, which are regularly scheduled visits with primary care providers (PCP) to screen for development and growth. As a result, most ASD screening occurs at primary health care visits for children (2).

However, there is a gap in advice for those receiving care at safety-net clinics. These clinics are places that provide healthcare to uninsured and other vulnerable populations, regardless of their ability to pay (3). State health insurance through Medicaid is available to all children born in the United States. Yet, people born outside of the United States often do not qualify for state-funded health insurance and therefore frequently access healthcare through safety-net clinics (4). In a rural Hispanic community that has access to safety-net clinics, patients still felt their preventative health needs were not being met (5). Thus, infrequent access to preventative healthcare for uninsured families could create a knowledge gap on general preventative services such as ASD screening.

Past literature has shown how screening correlates to more diagnoses of ASD among different socioeconomic statuses. In the United States, ASD rates are higher in families with higher socioeconomic status (6). However, in countries like Sweden with universal access to healthcare, lower socioeconomic status correlated with higher rates of ASD (7). This discrepancy suggests that in the United States, children of higher socioeconomic status receive appropriate ASD screening, while those of lower socioeconomic status may be falling through cracks in ASD identification and care.

With an understanding of the healthcare disparities associated with ASD, this project aimed to assess parental self-rated knowledge of ASD screening in a free clinic setting, with the goal of discerning the potential gaps in healthcare for this patient population.

## 2 Methods

During May through December 2022, participants were recruited from JayDoc Free Clinic, a free, student-run safety-net clinic affiliated with the University of Kansas School of Medicine in Wyandotte County, Kansas City, Kansas. Patients were recruited from the JayDoc’s walk-in and specialty clinics on Mondays, Tuesdays, and Wednesdays from 5 to 9 p.m.

Patients who had a child under 18 and were willing to participate in the study were given a two-part form, available in English and Spanish, to fill out and hand back at the end of their visit. The form contained a demographics survey including zip code, county of residence, ethnicity, marital status, education level, employment status, household language, insurance status, household income, number of children, and primary care provider status of their children. It also contained a Likert questionnaire of eight questions written by the study authors about the patient’s self-perceived knowledge of ASD and the importance of ASD screening. Response options included strongly agree, agree, disagree, strongly disagree, and not sure. Participants were not offered compensation and assured that their choice to participate would not affect their care. This project was approved by the University of Kansas IRB for a quality improvement (QI) study.

Following data collection, responses were categorized into positive (strongly agree, agree), negative (strongly disagree, disagree), and unsure. After data collection, the authors chose three questions for data analysis to focus the study on the impact of PCPs on ASD awareness. The authors did not analyze the other five questions. Responses to the three questions were examined closely in association with the child’s PCP status. Analysis occurred via R-studio using Pearson’s Chi-square (ꭓ^2^) test for independence. All tests were ran assuming a significance level α = 0.05.

## 3 Results

Of 73 participants who initiated surveys, 52 completed at least one Likert response. Of the 52 participants, 29 (55.8%) reported their child had a PCP. 69.8% of patients selected Wyandotte County, Kansas as their county of residence. Most surveyed identified as Hispanic or Latino (*n* = 39, 75%) and selected Spanish as their primary household language (*n* = 32, 61.5%). Additionally, 59.6% of participants reported their insurance status as “uninsured/none.” However, this question had a 26.9% non-response rate. Demographic data is reported in Table 1.

**Table 1:** Demographic data.

Baseline characteristic	PCP		No PCP		Full sample	
	*n*	%	*n*	%	*n*	%
**County of residence**					52	
Wyandotte (KS)	18	62.1	12	52.2	30	57.8
Johnson (KS)	4	13.8	5	21.7	9	17.3
Jackson (MO)	1	3.4	1	4.4	2	3.8
Other	2	6.9	0	0.0	2	3.8
Did not respond	4	13.8	5	21.7	9	17.3
**Race/ethnicity**					52	
White	1	3.4	1	4.3	2	3.8
Hispanic or Latino	20	69.0	19	82.6	39	75.0
Black or African American	4	13.8	3	13.0	7	13.5
Native American or American Indian	0	0.0	0	0.0	0	0.0
Asian/Pacific Islander	3	10.3	0	0.0	3	5.8
Other	1	3.4	0	0.0	1	1.9
**Health insurance status**					52	
Private	3	10.3	0	0	3	5.8
Medicare	2	6.9	1	4.4	3	5.8
Medicaid	1	3.5	0	0	1	1.9
None/uninsured	16	55.2	15	65.2	31	59.6
Did not respond	7	24.1	7	30.4	14	26.9
**Language**					52	
English	11	37.9	4	17.4	15	28.8
Spanish	14	48.3	18	78.3	32	61.5
Both	2	6.9	1	4.3	3	5.8
Other	2	6.9	0	0.0	2	3.8
**Child’s PCP status**					52	
PCP	–	–	–	–	29	55.8
No PCP	–	–	–	–	23	44.2

**Figure 1:**
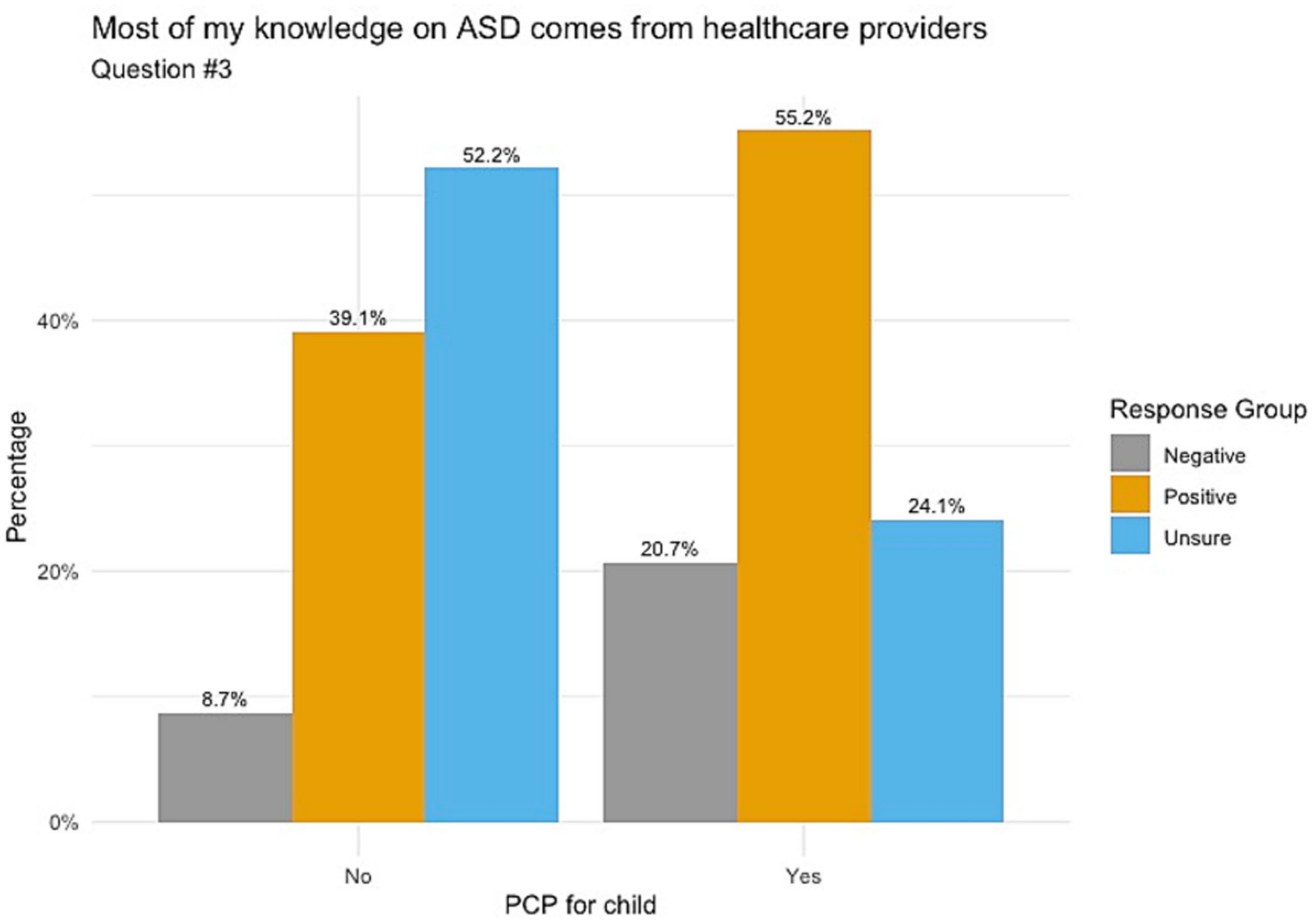
Knowledge of ASD from healthcare providers correlated with child’s PCP status. Non-significance between these groups was found with a *p*-value of 0.098.

**Figure 2:**
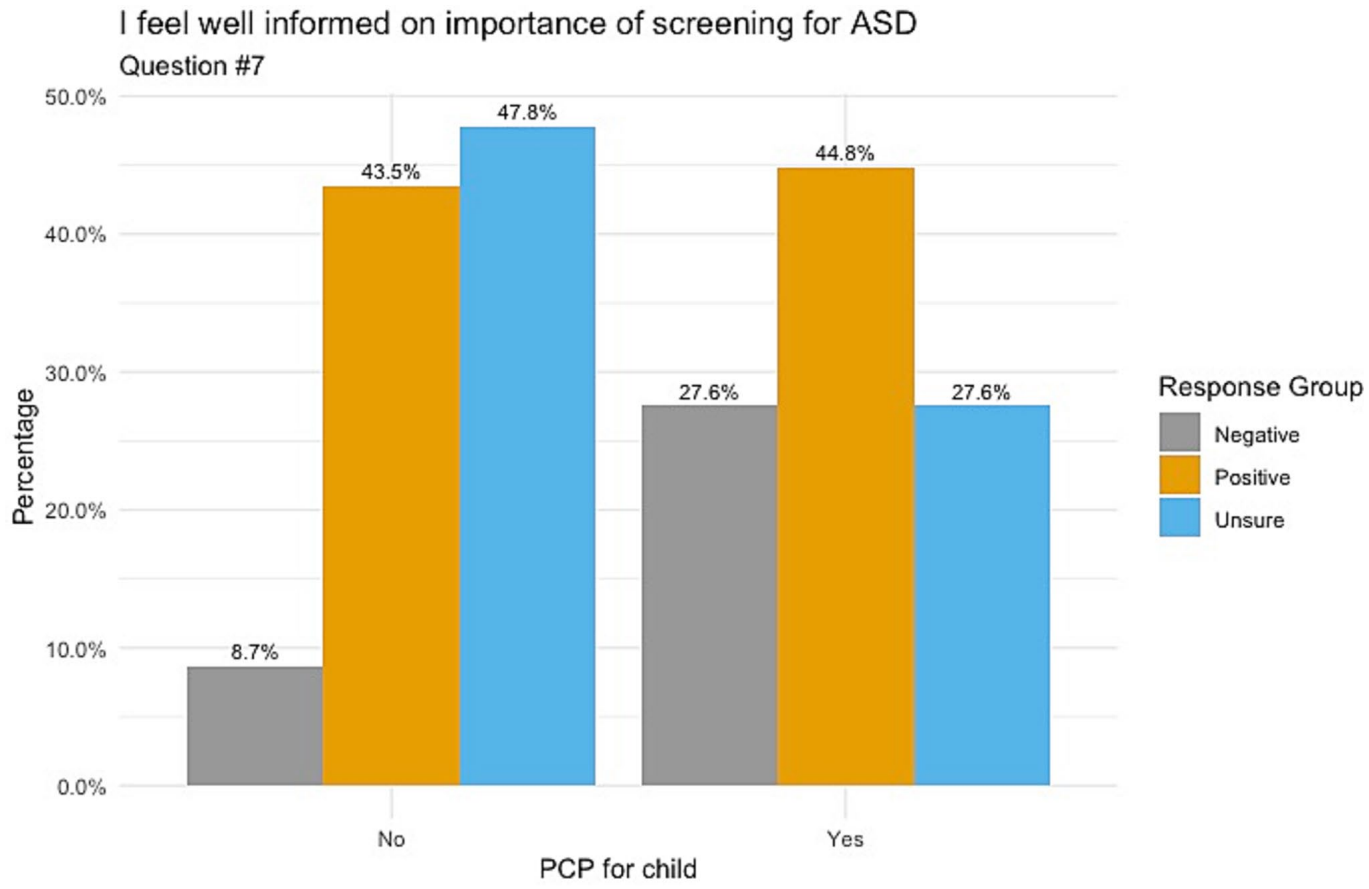
Feeling informed on the importance of ASD screening correlated with child’s PCP status. Non-significance between these groups was found with a *p*-value of 0.148.

**Figure 3:**
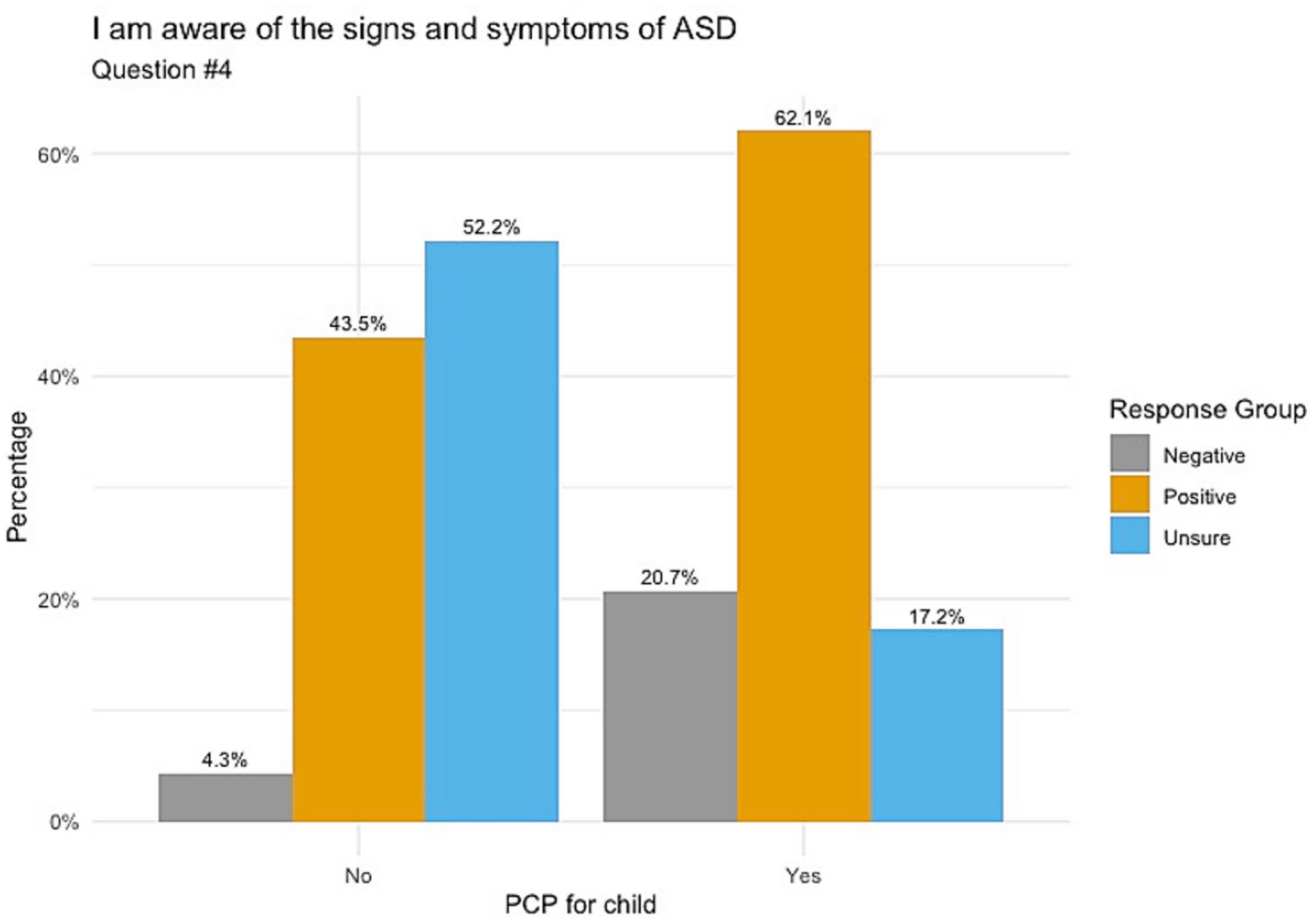
Awareness of signs and symptoms of ASD correlated with child’s PCP status. Significance between these groups was found to give a *p*-value of 0.017.

When responding to whether participants’ knowledge on ASD comes from healthcare providers, Figure 1 indicates those whose children do not have a PCP chose mostly unsure responses, followed by positive and negative responses (52.2, 39.1 and 8.7%, respectively) with a *p*-value of 0.098. Responses for understanding the importance of ASD screening showed that those with no PCP had balanced responses between unsure (47.8%) and positive (43.5%), as shown in Figure 2. Those with a PCP responded with more positive responses overall (44.8%) with even balance between negative and unsure responses (27.6% each) with a *p*-value of 0.148. Finally, the child’s PCP status was examined in relation to parental awareness of the signs and symptoms of ASD. For this, those without a PCP reported mostly unsure and positive responses (52.2 and 43.5%, respectively). For those with a PCP there was a 62.1% rate of positive responses, resulting in a significant difference between the groups of PCP vs. no PCP (*p* = 0.017), reported in Figure 3.

## 4 Discussion

Overall, these results indicate adults with children who access safety-net clinics feel unsure about their knowledge of ASD and autism screening, particularly if their child lacks a PCP. As a result, their children may be missing out on this crucial screen, resulting in a lack of access to services.

Given the intrinsic nature of safety-net clinics in caring for underserved populations, it is not surprising that 59.6% of participants lacked health insurance, and 26.9% of participants left the question blank altogether. The insurance status of the participants may help explain why only 55.8% of participants reported having a PCP for their child. While children are generally more likely to have health insurance than their parents, socioeconomic barriers affect whether children can access healthcare even with insurance (8). Those of low socioeconomic status are more likely to rely on safety-net clinics for primary care needs than traditional physician offices (9). Thus our finding that many participants did not have a PCP for their child points to the socioeconomic circumstances that lead people to safety-net clinics such as JayDoc. Additionally, the percentage of children with a PCP at JayDoc Clinic (55.8%) was not significantly different than that of the United States (47.0%) or Kansas (51.9%) as a whole (10).

The balance of positive responses with negative and unsure responses to questions about ASD could reflect lack of ASD knowledge and may relate to healthcare inaccessibility. Additionally, the significant association between a lack of PCP and unsure responses for understanding ASD signs and symptoms points to the importance of providers in promoting awareness and screening. This finding is in accordance with a previous study conducted in a safety-net clinic that examined barriers to colorectal cancer screening and found that provider communication impacts frequency of screening (11). While safety-net clinics can sometimes serve as PCPs, there is often discordance in the services they can typically provide. JayDoc, for example, is not able to provide longitudinal care to patients with the same healthcare provider due to rotating volunteer physicians. Benefits of PCPs, as indicated by our findings, include increased awareness of ASD among parents.

Limitations of this study include generalizability to populations outside the United States. Insurance status in the United States for adults under age 65 depends on citizenship and employment. Lacking health insurance makes regularly accessing healthcare unaffordable for many, causing these people to access healthcare through safety-net and free clinics. Many other countries have systems that prevent this care gap which could lead to better access to primary care providers. Another limitation is that survey distribution depended on cooperation from front-office staff. Dependence on front-office staff may have led to bias and consistency in the distribution of the surveys. Thirdly, of the 73 patients surveyed, 52 respondents submitted at least one answer. This lack of response suggests that some respondents missed a large portion of the survey due to printing on two sides of the paper or needing more time to complete the survey. Finally, the authors conducted this survey in Wyandotte County, Kansas, which ranks at 103rd out of 104 counties in Kansas for health outcomes, including life expectancy (12). These differences in health outcomes may be due to a lack of health literacy. The lack of awareness regarding ASD may be from poor health literacy in addition to poor access to primary care.

Our findings demonstrate that the assessment of PCP status of patient’s children could be helpful in guiding referrals to PCPs from safety-net clinics. Most patients who are seen at JayDoc are adults, thus JayDoc primary care referrals are currently focused on adult providers. The discovery that a significant number of participants lacked a designated primary care physician for their child, coupled with the observation that pediatric PCPs enhance ASD awareness, underscores the importance of advocating for referrals to pediatric PCPs. Asking about patients’ children and assessing socioeconomic barriers to PCPs could improve referral to pediatric providers. Additionally, ASD screening and education at JayDoc could be valuable, particularly for children without a PCP.

## Data availability statement

The raw data supporting the conclusions of this article will be made available by the authors, without undue reservation.

## Author contributions

RS and MO’D contributed to conception and design of the study. MO’D created the data-file. RS performed the statistical analysis. FB provided significant guidance throughout the study. RS, MO’D, and FB wrote the first draft of the manuscript. All authors contributed to the article and approved the submitted version.
